# The flying buttress construct for posterior spinopelvic fixation: a technical note

**DOI:** 10.1186/1748-7161-6-6

**Published:** 2011-04-13

**Authors:** Barend J van Royen, Martijn van Dijk, Dirk PH van Oostveen, Bas van Ooij, Agnita Stadhouder

**Affiliations:** 1 Department of Orthopaedic Surgery VU University Medical Center, De Boelelaan 1117, 1081 HV Amsterdam, The Netherlands; 2 Department of Orthopaedic Surgery, St. Antonius Hospital, Nieuwegein, The Netherlands

## Abstract

**Background:**

Posterior fusion of the spine to the pelvis in paediatric and adult spinal deformity is still challenging. Especially assembling of the posterior rod construct to the iliac screw is considered technically difficult. A variety of spinopelvic fixation techniques have been developed. However, extreme bending of the longitudinal rods or the use of 90-degree lateral offset connectors proved to be difficult, because the angle between the rod and the iliac screw varies from patient to patient.

**Methods:**

We adopted a new spinopelvic fixation system, in which iliac screws are side-to-side connected to the posterior thoracolumbar rod construct, independent of the angle between the rod and the iliac screw. Open angled parallel connectors are used to connect short iliac rods from the posterior rod construct to the iliac screws at both sides. The construct resembles in form and function an architectural Flying Buttress, or lateral support arches, used in Gothic cathedrals.

**Results and discussion:**

Three different cases that illustrate the Flying Buttress construct for spinopelvic fixation are reported here with the clinical details, radiographic findings and surgical technique used.

**Conclusion:**

The Flying Buttress construct may offer an alternative surgical option for spinopelvic fixation in circumstances wherein coronal or sagittal balance cannot be achieved, for example in cases with significant residual pelvic obliquity, or in revision spinal surgery for failed lumbosacral fusion.

## Introduction

Posterior fusion of the spine to the pelvis in paediatric and adult spine deformity poses many challenges to the spine surgeon. Spinopelvic fusion is indicated in neuromuscular scoliosis with a pelvic obliquity of more than 15 degrees, stabilizing adult fixed lumbosacral coronal plane curve, reduction of high grade spondylolisthesis, spinal pseudarthrosis (Charcot spine), surgical treatment of sacral tumours requiring (partial) sacrectomy, or as a tool to extend failed lumbosacral fixation [1-4]. A solid spinopelvic fusion can be achieved by a rigid posterior spinopelvic fixation that includes posterior lumbosacral spinal instrumentation with extension to the ilium [4].

The original surgical technique of posterior spinopelvic fixation has been developed by Allen and Ferguson in the 1980s [5-7]. They introduced smooth bended iliac rods that included the posterior instrumentation and the pelvis for the treatment of idiopathic adolescent scoliosis and revision scoliosis. The rods were inserted from the posterior superior iliac spine into each ilium between the inner and outer tables and extended within the ilium into the region above the iliac notch (the Galveston technique). However, contouring the rods is technically demanding and frequently rod breakage or bone resorption around the iliac rods has been reported [6,8]. Since then, a variety of spinopelvic fixation techniques have been developed including the use of bilateral iliac screws [1,9-13]. The use of long iliac screws with a 90-degrees offset connector between the iliac screw and rod, avoids complex lumbopelvic 3-dimensional rod bends. Peele et al. [14] showed that the use of iliac screws in neuromuscular spinal deformity corrections has similar results compared to the Galveston system, but there were fewer complications. Though, the posterior instrumentation technique for spinopelvic fixation is still a controversial topic [4].

**Figure 1:**
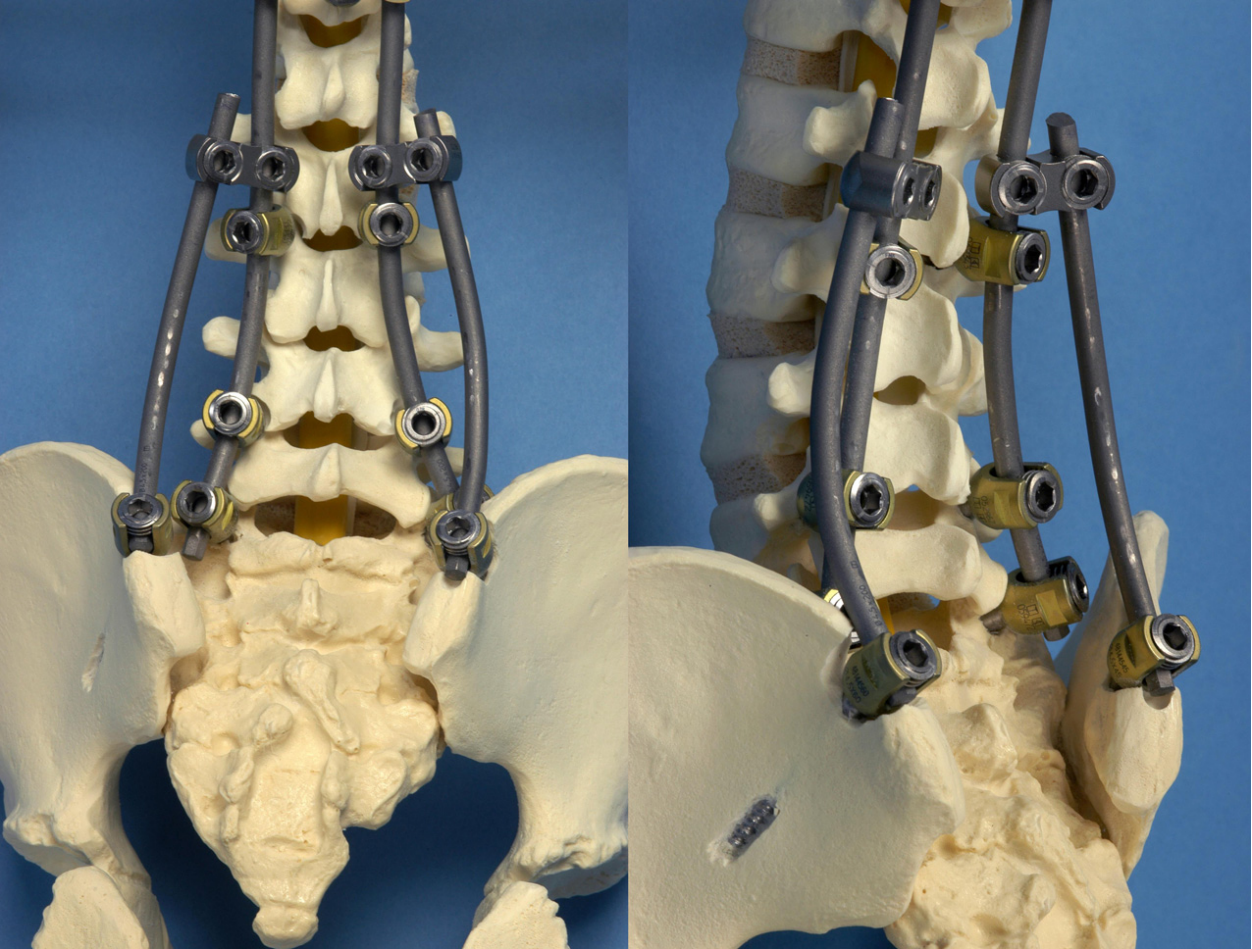
**1a and 1b: Model of a spine, showing the iliac screw position and rod placement in a flying buttress (FB) construct**.

In our experience, however, the assembly of the rod to the iliac screws itself or via a 90-degrees lateral offset connector proved to be technical difficult, because the angle between the rod and the iliac screw varies from patient to patient. We have devised a new spinopelvic fixation system, in which iliac screws are side-to-side connected to the posterior thoracolumbar rod construct, independent of the angle between the rod and the iliac screw (Figure 1). The construct resembles in form and function an architectural flying buttress (FB) used in Gothic cathedrals. Many famous Gothic cathedrals have FB constructs, or lateral support arches that 'fly' from the tops of the outside walls to large piers standing away from the building.

To our best knowledge, a side-to-side construct, or FB construct, for spinopelvic fixation has never been reported before in literature. The purpose of this study was to describe the new FB construct for spinopelvic fixation. Three cases that illustrate this procedure are reported here with the clinical details, radiographic findings and surgical technique used.

### Surgical technique

The patient is positioned in prone position on a Wilson spinal surgery frame (Orthopedic Systems, Inc., Union City, CA). A standard midline posterior approach to expose the thoracic, lumbar and sacral spine is performed. Polyaxial and/or monoaxial pedicle screws (Xia spinal system, Stryker Spine, Cestas, France) are connected to two long rods up to L5 or the sacrum as part of the deformity correction.

Using the same approach, the posterior superior iliac spine of the pelvis is exposed bilaterally subcutaneously over the long posterior spinal muscles. In this way, the sacral insertion of the erector spinae muscles is left untouched. The right and left posterior iliac crest from the posterior superior iliac spine is identified. The outer table of the ilium is exposed subperiosteal with a Cobb periosteal elevator, until the ischiatic notch can be palpated with the index finger. The ischiatic notch is used as a reference point for the screw direction. The entry point of the screw is chosen approximately 2 cm superior to the posterior superior iliac spine at the anterior side of the iliac crest. The iliac apophysis and a small amount of the iliac crest is removed with a rongeur to allow the screw head seated deeply in the posterior superior iliac spine. The blunt pedicle finder is introduced between the two tables of the ilium aiming 1 to 2 cm cranial of the ischiatic notch to the anterior superior iliac spine [15]. A ball-tipped probe with or without fluoroscopy can be used to confirm the path and to measure the depth. Bilaterally, a long poly-axial iliac screw is inserted in the same direction. The appropriate size of the screw is depending on the anatomy of the iliac spine and can be up to 100 mm long and 8.5 mm in diameter. Two parallel iliac rods are bended and inserted bilaterally into the iliac screws after tunnelling anterior under the long posterior spinal muscles. Connection of the iliac rods with the spinal longitudinal fusion rods is accommodated by open low-profile parallel connectors, with different angles (Figure 1) and fixed with a blocker. Allografts from our local bone bank or synthetic bone graft substitutes were used to achieve a solid spinopelvic fusion. Closure of the wound and skin was performed with a vacuum drain for 24 hours.

### Illustrative cases

#### Case 1

**Figure 2:**
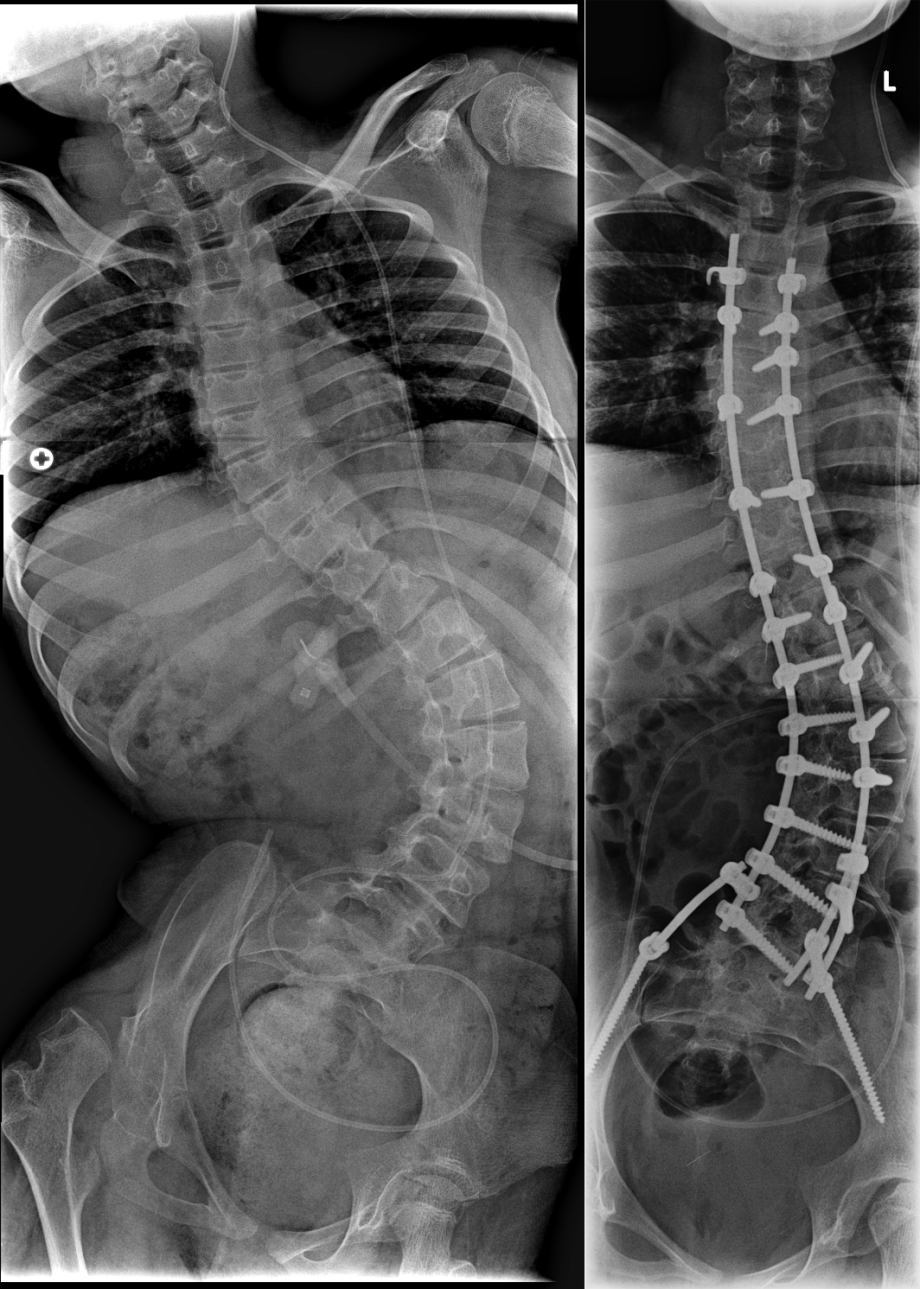
**Case 1. Anteroposterior radiographs made preoperatively (Figure 2a) and following posterior instrumentation from T3 to the pelvis (Figure 2b) in a 8-year old patient with a severe neuromuscular scoliosis**.

A 8-year old wheelchair depending girl with spastic cerebral palsy, congenital hydrocephalus and epileptic episodes presented with a severe progressive neuromuscular thoracolumbar scoliosis and pelvic obliquity (Figure 2a). Her weight was 30 kg. She experienced increasing problems in her sitting balance. Radiographs showed a 93 degree Cobb-angle T11-L4. Treatment consisted of posterior only scoliosis correction with multisegmental screw fixation and a 4.5-mm titanium alloy double-rod instrumentation combined with a posterior arthrodesis from T3 to the pelvis. Two iliac screws were placed and connected to the posterior rod construct by using a FB construct. A complete correction of the scoliotic deformity was not feasible, and an asymmetrical lumbopelvic angle remained. As a result, the two iliac rods of the FB construct were asymmetrically connected to the posterior rod construct by respectively a 25 degree parallel connector at the right site and a 0 degree parallel at the left site (Figure 2b). Postoperatively, the patient was treated at the pediatric intensive care unit for 1 day and recovered uneventful. There were no postoperative complications. At 4 years follow-up, she is in a good condition, the spine shows a solid fusion without rod or screw failure.

#### Case 2

**Figure 3:**
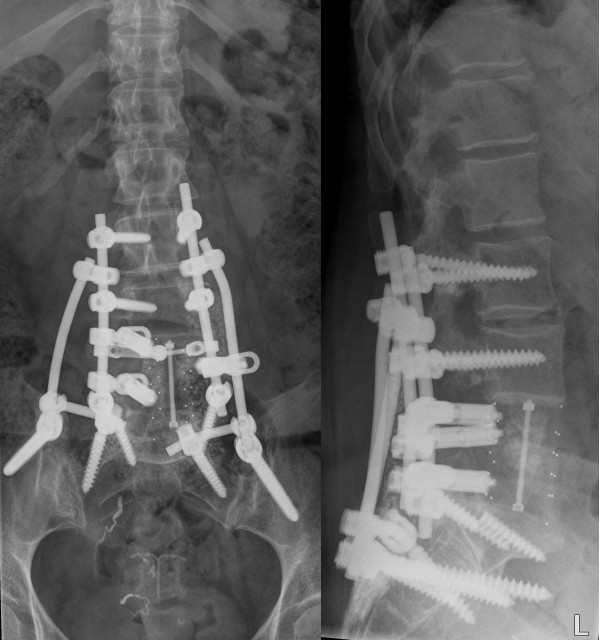
**Case 2. A 42-year old patient with a pseudarthrosis of L5-S1 and failure of both S1 screws following en block spondylectomy for a chordoma of L5 who underwent extension of the posterior fusion with lumbosacral and spinopelvic fixation**. Figure **3a**: Postoperative anteroposterior radiograph. Figure **3b**: Postoperative lateral radiograph

A 42-year old female was operated in 2005 for a chordoma of L5. Patient was treated with a combined posteroanterior en bloc excision of L5. Posterior pedicle screw instrumentation was performed from L3 to S1. For anterior column reconstruction a carbonfiber stackable cage filled with tricalcium phosphate granulate and allogenic bone-grafts was placed. At two-year follow-up, she developed progressive low back pain, due to pseudarthrosis of L5-S1 and subsequently failure of both S1 screws. Revision surgery was performed by additional posterior iliac screw fixation combined with sacral alar screws in a FB construct (Figure 3). The sacral alar screws were fixed via a 90-degrees lateral offset connector to the FB construct. In addition, posterior allogenic bone grafting was applied. A rigid posterior lumbosacral and spinopelvic fixation was achieved. Postoperative there were no complications and mobilization was possible immediate after surgery without external support. Despite some complaints of intermittent low back pain without any radiculopathy, the construction is solid with no radiographic signs of tumour recurrence or hardware-related complications observed at 3 years follow-up.

#### Case 3

**Figure 4:**
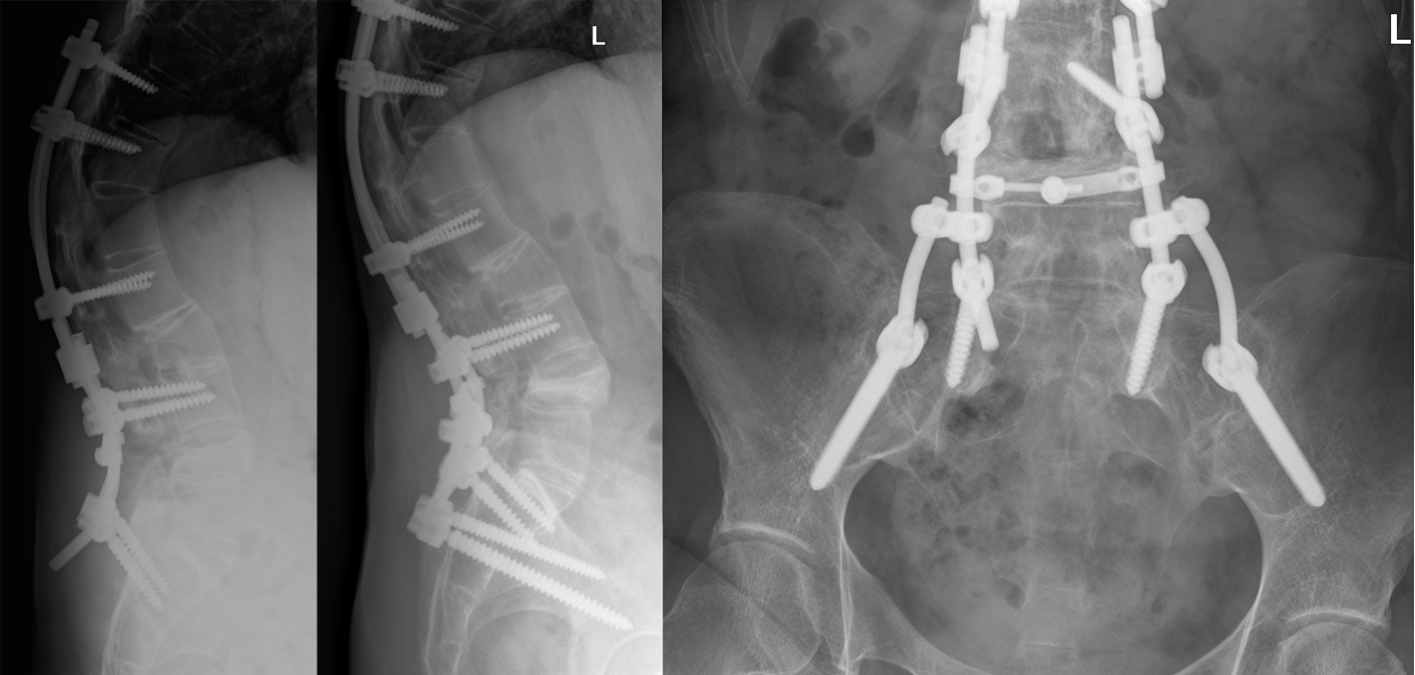
**Case 3. A 44-year old woman with a progressive thoracolumbar kyphotic deformity caused by ankylosing spondylitis was treated by a 40 degree closing wedge lumbar osteotomy at level L4**. Unfortunately, the pedicle screw fixation in S1 broke out, resulting in loss of deformity correction at L4. Revision surgery was performed to restore the deformity correction with bilateral iliac screws in a FB construct. Figure 4a: Pre-operative lateral radiograph. Note the loss of correction and S1 screw failure. Figure 4b: Postoperative lateral radiograph. Figure 4c: Postoperative anteroposterior radiopgraph.

A 44-year old woman with a 19-year history of ankylosing spondylitis presented with a progressive thoracolumbar kyphotic deformity. A 40 degree closing wedge lumbar osteotomy was performed at level L4, with pedicle screw fixation T10-S1. Postoperative plaster immobilization with a TLSO with one leg included was used for three months. Unfortunately, the pedicle screw fixation in S1 showed a breakout, resulting in loss of deformity correction at L4 (Figure 4A). Revision surgery was performed to restore the deformity correction and re-fixate the osteotomy. The patient was placed in prone position and the loss of deformity was corrected by extending the surgical table. The revised correction was re-fixated with bilateral iliac screws in a FB construct and additional posterior bone grafting (Figure 4B and 4C). The spontaneously repositioned sacral screws were left in place knowing that they did not add any stability to the construct in the osteoporotic bone. At two years follow-up, the radiographs showed an unaltered reduction with no loss of correction and complete fusion.

## Discussion

In spinopelvic instrumentation, the assembling of the iliac screws to the longitudinal posterior rod construct remains technically challenging in spinal deformity surgery. This is especially the case in patients with severe neuromuscular scoliosis and pelvic obliquity wherein complete correction of the scoliotic curve could not be achieved. In these cases an asymmetrical postoperative lumbosacral angle makes the assembly of the posterior rod to the iliac screws technically difficult. Alternatively, bending of the longitudinal posterior rod, sometimes in extreme angulations, to connect the rod to the iliac screw at both sides may be technically very demanding. In addition, the currently often-used titanium alloy rods do not allow severe bending because of the risk of postoperative rod breakage.

The instrumentation of the FB construct for spinopelvic fixation, in contrast, is easy to apply and overcomes these technical difficulties. The advantages of the FB construct for spinopelvic fixation are the use of a familiar surgical technique and a short learning curve. Spine surgeons who are comfortable with the use of iliac screws and or the Galveston technique for spinopelvic fixation can readily adopt this FB construct. The most important benefit of the use of this FB construct in deformity surgery is that the thoracolumbar deformity can be instrumented and corrected first, without taking into consideration how to assemble the longitudinal posterior rods to the iliac screws. By using open (angled) parallel connectors, the two iliac rods are easily linked from the iliac screws to the longitudinal posterior rod construct. In this way, these parallel connectors can be easily fixed to the longitudinal rod between two already placed and tightened pedicle screws, independent of the lumbar lordosis or residual pelvic obliquity.

As a consequence, the FB construct may offer an alternative surgical option for spinopelvic fixation in circumstances wherein complete correction of the scoliotic curve cannot be achieved and significant residual pelvic obliquity persists. This is most often the case in patients with a severe rigid neuromuscular scoliosis, as shown in case 1. Both iliac rods are simply placed and subsequently connected to the posterior thoracolumbar screw-rod construct of the residual scoliotic deformity in an asymmetrical FB construct.

In case the placement of more than one iliac screw on each side is indicated to provide a higher construct stability, for example in unstable situations caused by total sacrectomy [16], assembling of the iliac rod to the two ipsilateral iliac screws in a FB construct can also be performed easily. If necessary, a cross connector can be placed between the longitudal rod and the iliac rod to even more improve the rigidity of the FB construct.

Finally, the FB construct offers an attractive surgical option in revision spinal surgery for failed lumbosacral fusion, as shown in case 2 and 3. In case 2, pseudarthrosis between the cage and S1 resulted in screw breakage of the S1 screws. Unfortunately, re-placement of the broken S1 screws was not possible. Therefore, two additional sacral alar screws were placed into the lateral anterior cortical bone of the sacrum. Adding iliac screws and extending the longitudinal rod fixation to the sacrum and ilium in a FB construct did achieve a rigid fixation. By using the FB construct in revision spinal surgery, no extensive surgical dissection is necessary to add the FB construct to the existing longitudinal rod construct. In addition, the use of open parallel connectors avoids the need to temporarily loosen the pedicle screw-rod fixation to connect the iliac rods. In case 3, the osteoporotic bone quality of the sacrum could not withstand the high kyphotic forces after lumbar osteotomy in ankylosing spondylitis. This resulted in breakout of the sacral screws with loss of correction as a result of unfavourable biomechanics of a great lever arm at the lumbosacral junction and severe osteoporosis of the sacral bone. Revision of the lumbar osteotomy with extension of the instrumentation to the sacrum was easily achieved by using iliac screws and open parallel connectors in a FB construct. Obviously, primary instrumentation and fixation to the pelvis in a FB construct after lumbar osteotomy in ankylosing spondylitis with severe secondary osteoporosis will prevent this complication in the future.

From a biomechanical point of view, the half arched side-to-side spinopelvic fixation is a very strong construct to transfer lateral thrust forces from the spine to the iliac crest. The mechanical philosophy and form of this construct, however, is not new. The Romans invented the lateral thrust arch, and in doing so, created the origin for the FB in Gothic cathedrals. In the Gothic architecture, a FB is known as an open half arch that gives extra support to the upper part of a wall by transmitting the thrust of a vault or roof to a support that stand outside. In this way, the load placed on the top of the arch is transferred along the curve of the arch to the sides, and thus down to the foundations. The spinopelvic fixation technique presented here resembles in form and mechanical function such an architectural FB used in many Gothic cathedrals, for example the Notre Dame Cathedral in Paris, France.

Although the iliac tables of the hemipelvis have proven to be a save and solid foundation for rigid spinopelvic fixation, this technique requires fixation across a normally mobile sacroiliac joint. Obviously, this is not the case in ankylosing spondylitis, where there is no motion in the complete fused sacroiliac joints (case 3). However, in all other cases, instrumentation anterior to the posterosuperior corner of S1, the lumbosacral pivot point[4,17], and additional posterolateral sacroiliac bone grafting is essential to create a solid spinopelvic fusion. In addition, anterior lumbosacral interbody fusion may be considered in some indications.

In conclusion, the flying buttress (FB) construct may offer an alternative surgical option for spinopelvic fixation in circumstances wherein coronal or sagittal balance cannot be achieved, for example in cases with significant residual pelvic obliquity, or in revision spinal surgery for failed lumbosacral fusion. The instrumentation of a FB construct for spinopelvic fixation has the advantage of modularity and is easy to assemble. The FB construct is a solid fixation that shows mechanical similarity with lateral thrust arches of the Gothic architecture. However, biomechanical testing and long-term follow-up of the FB construct for spinopelvic fixation is needed.

## Competing interests

The authors declare that they have no competing interests.

## Authors' contributions

BvR set the theoretical bases and conceived the surgical procedure, performing all of the operations and coordinated the preparation of the manuscript; MvD and DvO analysed and interpreted the patient data, and were major contributors in writing the manuscript. BvO did research and helped to list references; AS helped to draft the manuscript and revised it critically. All authors read and approved the final manuscript.
